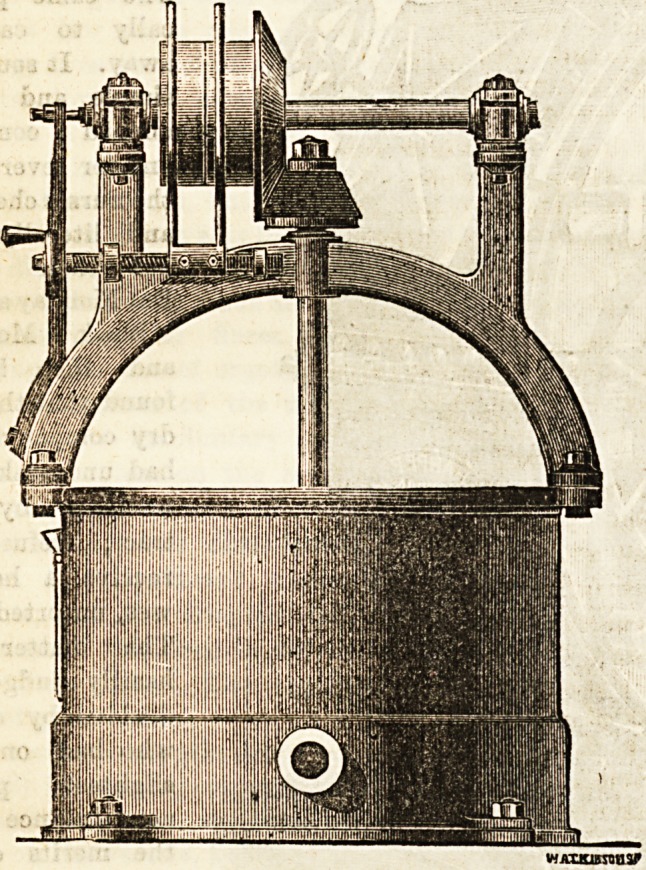# The Laundry

**Published:** 1892-09-17

**Authors:** 


### THE INSTITUTIONAL WORKSHOP.

### PRACTICAL DEPARTMENTS.

### THE LAUNDRY.—II.

THE washing machine here shown is a large one, which is doing an immense amount of work every day, and accomplishing it so satisfactorily that it is in high favour in the well-ordered laundry at Whitechapel Infirmary. It is also used for rinsing and blueing, and is known as "Twelvetree's Patent Rotary Washer." It has the automatic reversing gear, and the outer shell is made of stout steel plates, and the inner cage of galvanised tubes, so contrived as to lift the clothes out of the water at every revolution. Messrs. Harper Twelvetrees, 8, City Road, have also furnished the laundry, worked entirely with hand-power machines, at the Hospital for Incurable Children in Cheyne Walk, and the contrast in size of the "washers" in the latter place with the giant at Whitechapel Infirmary is rather remarkable; but each is suited for the work required of it.

The great laundry at the London Hospital was arranged and fitted by Messrs. Bradford, of High Holborn, some eighteen years ago, and it speaks well for the machines that they have actually done continuous work ever since. We must remember, too, the amount of linen which passes through that laundry regularly, for the sheets are numbered by thousands, and the blankets and shirts by hundreds. There is a separate department for the staff, and 300 nurses' dresses form one item in the week's work. Wringers are of various kinds—some apart from, and others attached to, washing machines—and they free the clothes from water and soap in very swift fashion.

When linen which has been used for "an infectious case" in a hospital is removed from the patient's bedside, in a covered zinc pail, in which carbolic lotion is previously placed, it is either carried away to "the shoot," expressly devised for its transfer (apart from all other linen), or it is conveyed at once to the basement and placed in the tank dedicated to it. In this it is left to soak in carbolic, of which there is a sufficient quantity to entirely cover, not merely to damp the articles, until the following day, when it is fished out with a suitable implement, and passed through a wringer fixed close beside the tank, and thence transferred to the laundry, either on the premises or elsewhere, to go through the ordinary routine. This may be thought a matter-of-course arrangement hardly demanding mention, but like many other modern customs it is the outcome of much discomfort. We can look back five and twenty years and remember the accumulation of soiled linen, hoarded for a week in the underground room of a children's hospital, and on the day devoted to the gruesome task, it was all sorted and counted by the unfortunate personage responsible in the matter. It does not need any uncommon stretch of imagination to depict some of the odours in that "chamber of horrors." And yet one more scene comes to our mind. Some twelve years back, when antiseptics were obtaining favourable attention, and thoughtful people realised that foul linen harboured many a germ, the decree went forth in a certain small hospital that all linen was to be immersed in large tanks of carbolic pending the arrival of the laundry man, who came periodically to carry it away. It sounded a simple and easily-obeyed command, and for several days the nurses cheerfully and literally acted out their orders. But Monday arrived, "Black Monday," and then it was found that the laundry company which had undertaken the washing, by contract, refused to receive a heap of wet, unsorted linen. The matter was hastily judged and decided by officials who had only the slightest possible acquaintance with the merits of the case, and they issued a rule which practically amounted to this. The laws of health were to be studied, and therefore the carbolic tank was to be in regular use, but the laundryman's wishes were also to be considered, and when the water had been drained off the linen, previous to his coming, it was to be received by a nurse (she was a trained and refined woman, by the way), who was to take out and count before him every article in that damp chaos. What would not that unfortunate woman have given to be put in possession of the wringer as described above, and also to have had a chance of sharing in the many benefits of to-day, when able administration pervades laundries as well as the other departments of institution work. Practical heads of each branch are absolutely requisite, and nowhere is their value better realised than in the one which we are now discussing.

A small hand-wringer can also be fixed to a bath or other receptacle in which it has been found convenient to disinfect special linen, thus making it easy for the nurse to pass everything through the rollers into her laundry basket. Some simple contrivance of this sort will be found valuable in any ward set apart for cholera or other similar diseases, when it is above everything necessary for immediate disinfection to be arranged for. The whole thing would take so little floor space that it could always be fitted up at short notice in a bath-room or other chamber contiguous to the patient. Sanatoriums connected with schools should always contain a complete apparatus for the treatment of linen. Whether doubtfully or decidedly infectious, it is impossible to take too many precautions in the case especially of "preventable" diseases, and it should never be permitted for the clothes to be removed to any laundry until they have first been in some reliable way made absolutely safe by a competent person.

The Centrifugal Wringers certainly form a most interesting feature in laundry work, and there is something weirdly mysterious in their motion. It is so bewilderingly rapid, and when it ceases, and the articles packed in a few minutes before quite wet are removed from it dry, we feel an increasing respect for the fascinating Hydro-Extractor. There are many good ones, and we have here a sketch of the

"Top-driven" one, supplied by Messrs. Twelvetrees, and it merits praise, as do also the "Under-driven" and "Direct-driven" varieties. Most makers have the three kinds, and each one of them represents an enormous saving of time and of labour.

(*To be continued.*)

## Figures and Tables

**Figure f1:**
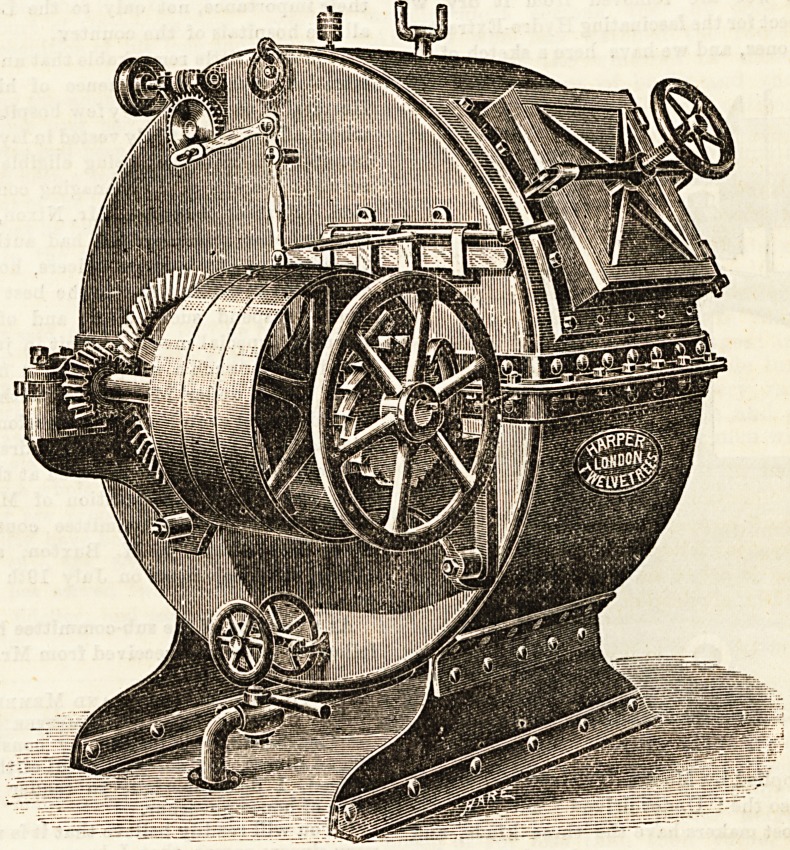


**Figure f2:**